# Beyond ethical and curricular guidelines in global health: attitudinal development on international service-learning trips

**DOI:** 10.1186/s12909-015-0357-7

**Published:** 2015-04-03

**Authors:** William B Ventres, Calvin L Wilson

**Affiliations:** 1 Institute for Studies in History, Anthropology and Archeology, University of El Salvador, San Salvador, El Salvador; 2 Department of Family Medicine, Oregon Health and Science University, Portland, OR USA; 3 Department of Family Medicine, University of Colorado Anschutz School of Medicine, Denver, CO USA; 4 Urbanización Buenos Aires III, Block H, Calle Los Maquilishuat, N° 3-A, San Salvador, El Salvador

**Keywords:** Attitude of health personnel, Bioethics, Engineering education, Global health, International educational exchange, Medical education, Nursing education, Public health education for professionals, Social responsibility

## Abstract

**Background:**

Health professionals from high-income countries are increasingly becoming involved in international service-learning trips in low and low/middle-income countries. While much has been written about the ethics and curricular guidelines related to such endeavors, scant attention has been paid to the attitudes with which trainees and clinicians enter into or return from them. In this essay the authors explore how attitudes contribute to the success or failure of international service-learning trips.

**Discussion:**

The authors submit that the attitudes with which visiting health professionals approach international service-learning trips are much more critical to the success of these experiences than their demonstrated knowledge base or specialized skill sets. They list five attitudes that can aid those participating in international service-learning trips. They list five troubling attitudes that, while common, those participating in international service-learning trips can learn to recognize and avoid. They suggest five strategies key to learning respectful attitudes that can foster success in such cross-cultural activities. Lastly, the authors review several concepts related to attitude development in short or long-term global health work.

**Summary:**

The attitudes with which visiting health professionals approach international service-learning activities can be important components of the success or failure of the experiences. Through thoughtful consideration of attitudes and approaches, participants on such trips can build a framework for rich and rewarding experiences in global medicine and global health.

## Background

Health professionals from high-income countries (HICs) are increasingly becoming involved in international service-learning trips (ISLTs) in low and low/middle-income countries (L/LMICs) [1]. Although the ethics of these interventions have been reviewed elsewhere [2-5], and standardized curricular approaches to global medicine are gaining strength [6-10], attitudes relevant to behaviors during and after ISLTs have received scant attention [11,12]. In spite of this deficiency, we submit that the attitudes with which visiting health professionals approach ISLTs are much more critical to the success of these experiences than their knowledge base or specialized skill set, regardless of level of training or health discipline.

We review a variety of attitudes crucial to the success or failure of any ISLT, basing this review on insights gained from many years practicing global medicine [13], teaching global health [14], observing the clinical work of other professionals internationally [15], and studying issues of health equity worldwide [16,17]. Drawing from our successes as well as our mistakes, we identify five attitudes that can help ISLT participants build collaborative bonds and another five problematic attitudes that participants can learn to recognize and avoid. We also suggest five key approaches to developing attitudes that foster mutual respect on ISLTs. Lastly, we review five concepts that inform our perspectives on attitude development. We believe these concepts are foundational elements of global health work for trainees and practitioners in any health profession or specialty (including those within the fields of medicine, nursing, public health, engineering, and the allied health professions).

## Discussion

### Attitudinal goals

Effective ISLTs are bidirectional in scope, combining service and teaching contributions of visiting health professionals with learning from local contexts and cultures. Given this dual reality, the following attitudes are essential to contributing to and benefiting from ISLTs in meaningful ways.

*Open-mindedness* implies a willingness to take note of how others perceive and act in international environments as well as to question the conventional values that form the basis of professional socialization in HICs. Moreover, open-mindedness recognizes that no one works in isolation, and that the search for health is a collective endeavor. Professionals of diverse skills work together with patients, families, and loved ones: each participant in the process brings unique perspectives to the healing relationship.

*Humility* emerges from the notion that all share in the challenges of the human condition and suggests that this commonality outweighs differences in professional skill, ethnic background, wealth, or social standing. Humility recognizes that different points of view are worthy of thoughtful consideration and that “reality” changes along with perspectives. Appreciating this means paying careful attention to the historical, political, linguistic, and economic contexts at play around the world. Humility is especially important when working with people who have been oppressed because of the color of their skin, their lack of economic resources and gainful employment, the place of their birth, or any one of many other factors traditionally associated with discrimination or subjugation.

*Generosity*, like altruism, signifies a willingness to give of one’s time, resources, and self. In contrast to altruism, however, generosity implies a personal and professional give and take. Health professionals involved in global medicine and public health give to others through care and competence; in return they receive benefits born of reciprocal expressions of trust and respect. An attitude of generosity can result in mutual participatory involvement, which can in turn foster relational awareness and improve one’s ability to work across cultural boundaries.

*Patience.* Success in global health takes time. While short-term interventions can improve health outcomes if they are part of a larger, well-organized, and thoughtful process, they only set the stage for imagining progress. The shorter the time spent on an ISLT, the more likely that the participants rather than the hosts will benefit. The ability to stop, look, and listen, over time—developing relationships with people along the way—cannot be underestimated in global health work. Time is one thread that builds trust across many frontiers, including culture, geography, and social context.

*Excellence* means understanding that principles such as continuity, comprehensiveness, and person-centeredness play roles of equal importance to disease-focused diagnosis and treatment in the evidence-based practice of modern health care. By means of their biomedical training and, in many cases, profit-oriented models of reimbursement, health professionals in HICs often develop cognitive and therapeutic approaches successfully applicable only in settings of organizational and technological abundance. The concept of clinical excellence, in contrast, acknowledges that health professionals (at all levels of intervention and in all settings) must consider issues of local culture, variations in resource availability, and determinants of health in order to provide optimum care. Such perspectives enhance collective abilities to attend to those living in circumstances of economic poverty, social marginalization, and geographic inaccessibility.

### Attitudinal traps

Several attitudes negatively influence the inter-cultural effectiveness of professionals on ISLTs or other global medical and public health activities, and impede their own personal and professional growth back in their home culture.

*Arrogance* is marked by the desire to fulfill one’s own unconscious psychological need for significance and accomplishment. It often occurs in international settings when, owing to differences in material possessions, academic backgrounds, or political and historical circumstances, some people think they are better than others. Ironically, it can be abetted by the admiration and attentiveness of host colleagues. Arrogance is the antithesis of the humility described above.

*Hegemony* is the domination of a certain ideology, culture, or class over another. It is rooted in asymmetries of power and associated with the influence sociopolitical forces have on both interpersonal and group dynamics. It often manifests itself in attempts at control or conversion. Hegemony can be blatant; it is also often subtle, as when professionals from HICs condescendingly disparage other health care systems while failing to consider how geography, history, and politics limit health and well being in their home environments.

*Balkanization* occurs when health professionals on ISLTs cannot see the forest for the trees: they implement projects, temporarily staff clinics, and deliver medications in the absence of any organized consideration of context, environment, or cooperation. Given the challenges of organizing health care delivery in HICs, particularly in the United States, reproducing similar fragmented systems elsewhere makes little sense.

*Indebtedness* takes two forms. In the first, it implies that hosts should be grateful for what visitors bring. It can occur in interactions as simple as providing medical equipment or as complex as implementing comprehensive interventions. The result, however, is the same: “Look what I ‘gotcha’. Aren’t you thankful?” In a second, more subtle, yet more insidious meaning, indebtedness suggests domination. Because of what visitors bring, hosts become indebted: “I ‘gotcha’.” Both represent shadow sides of inappropriate gift-giving unfortunately widespread around the world.

*Power by proxy,* or “buddy” development, occurs when somebody knows somebody who knows somebody (who often knows somebody else) who helps the initial somebody set up an ISLT with the vague goal of helping people in settings of need. The bidirectional migration of medical and public health personnel around the world make this an increasingly common event. While friends are wonderful to have wherever one is, genuine global health development requires nurturance of collaborative, continuous, and dynamic relationships within host countries, not just imposing one’s presence or implementing a pet project at the arranged invitation of another.

### Teaching and learning attitudes

Ample literature on teaching and learning methods exists [18,19], yet the following five general approaches can enhance one’s abilities to be sensitive rather than insensitive, and adept rather than offensive, while participating on ISLTs. These approaches are valid at any stage of professional development or practice.

#### Be curious and inquisitive

Do not approach global medicine from a standpoint of expertise, but with a quest for exploration, taking into consideration the limits of one’s competence in unfamiliar situations [20]. This exploration should include both a willingness to question fixed beliefs and a commitment to listen deeply; such is the reflective process of examining external context and internal belief structure concurrently [21,22].

#### Prioritize learning

Don't know the language? Learn it. Unfamiliar with the geography or culture? Get out and about. Lacking understanding of the history or politics? Read articles and books, watch movies, and engage with informed colleagues closer to home prior to travelling abroad. Feeling bewildered while there? Listen for beliefs that motivate apparently confusing behaviors. In cultures different from one’s own, seek out shared commonalities while investigating the causes and effects of differences. Metaphorically “step into the skins” of others and try to see the world as they do. Acknowledge that clinical practices differ around the world, depending on variations in prevalence of disease, understandings of health, principles of treatment, and interpretations of cultural norms. Appreciate that the overall outcomes of these practices are often equal to or better than those in HICs [23].

#### Practice personal reflection

In addition to exploring similarities and dissimilarities between and within cultures, ask questions about one’s own personal and professional upbringing and “native” culture [24]. Be willing to see “new” realities in one’s home environment, realities frequently obscured by the uniformity of daily routines and the habitual presence of confounding judgments, assumptions, and internal emotional states [25].

#### Choose mentors and models wisely

Humanistic attitudes are part and parcel of high-quality health care practice [26]. Seek out those who demonstrate these attitudes, taking cues from them on how to successfully navigate the challenges of working across cultures. Conversely, be someone who is multi-dimensionally aware, responsible in the face of social inequity, and responsive to the uniqueness of individual differences.

#### Grow

Successful international work inevitably triggers personal growth. Questioning entrenched presuppositions, adapting to challenging conditions, and becoming conscious of global human interdependence are all essential educational dimensions of ISLTs. Welcome change within yourself.

### Education/development values

Our assessment of these attitudes and approaches is based on our experiences and beliefs about what global health development means. We try to hold and live up to the following tenets as guides to working across cultural and geographic borders.

#### Discovery is a shared process

In most situations, whether short or long-term, the most valuable lessons occur when all parties are interested in learning from each other. The best way to encourage this quality is to demonstrate it.

#### Knowledge goes both ways

Access to advanced diagnostic and therapeutic technologies is an important part of medicine in the 21^st^ century, but it is far from being the only part of authentic development. Wisdom comes in many forms; astute ISLT participants recognize it in their hosts and return home all the wiser for having done so.

#### International work prepares people for work at home

All too often, participants in ISLTs want to accomplish what they cannot in their countries of origin. However, ISLTs can also encourage participants to practice with and advocate for those people in increasingly diverse, multi-cultural HICs, including refugees, immigrants, and others living on the margins of society who may feel like strangers in a foreign land [27].

#### Recognizing context is key to success

ISLTs are destined to fail if their participants lack thoughtful consideration of local explanations of illness, the causes and effects of social determinants, and the influences of geography, politics, history, and economics on health and disease.

#### Self-examination is critically important

Examining individual and collective motivations, understanding personal aptitudes and attitudes, and exploring the edges of unconscious biases are all important steps toward growing the sense of self-in-relationship that is vital to making progress toward health [28].

## Summary

ISLTs can introduce health professionals in training and practice from HICs to the complexities of working in L/LMICs, provide grounded perspectives on the responsibilities inherent in global medicine and public health, and whet appetites for launching or expanding long-term careers in global health, both domestically and overseas. These projects offer health professionals a chance to explore “making a difference” in settings of significant need.

However, most of the “real” development from ISLTs does not happen to people in L/LMICs. It happens within those participating trainees and practicing health professionals as part of the process of reflecting upon the attitudes with which they enter into and return from such projects. These attitudes, the thoughts and feelings participants on ISLTs each carry with them, can be the makings of failure or the ingredients of success while abroad or back at home. Through thoughtful consideration of attitudes and approaches, ISLT participants can build a framework for rich and rewarding experiences in global medicine and global health.
